# NGS Reveals Molecular Pathways Affected by Obesity and Weight Loss-Related Changes in miRNA Levels in Adipose Tissue

**DOI:** 10.3390/ijms19010066

**Published:** 2017-12-27

**Authors:** Alina Kuryłowicz, Zofia Wicik, Magdalena Owczarz, Marta Izabela Jonas, Marta Kotlarek, Michał Świerniak, Wojciech Lisik, Maurycy Jonas, Bartłomiej Noszczyk, Monika Puzianowska-Kuźnicka

**Affiliations:** 1 Department of Human Epigenetics, Mossakowski Medical Research Centre, Polish Academy of Sciences (PAS), 5 Pawinskiego Street, 02106 Warsaw, Poland; zwicik@imdik.pan.pl (Z.W.); mowczarz@imdik.pan.pl (M.O.); martajonas@poczta.onet.pl (M.I.J.); 2 Genomic Medicine, Medical University of Warsaw, 02097 Warsaw, Poland; marta.kotlarek@wum.edu.pl (M.K.); michal.swierniak@wum.edu.pl (M.Ś.); 3 Department of General and Transplantation Surgery, Medical University of Warsaw, 00001 Warsaw, Poland; wojciech.lisik@wum.edu.pl (W.L.); morjon@poczta.onet.pl (M.J.); 4 Department of Plastic Surgery, Medical Centre of Postgraduate Education, 00416 Warsaw, Poland; noszczyk@melilot.pl; 5 Department of Geriatrics and Gerontology, Medical Centre of Postgraduate Education, 01826 Warsaw, Poland

**Keywords:** next generation sequencing, miRNome, adipose tissue, obesity, molecular pathways

## Abstract

Both obesity and weight loss may cause molecular changes in adipose tissue. This study aimed to characterize changes in adipose tissue miRNome in order to identify molecular pathways affected by obesity and weight changes. Next generation sequencing (NGS) was applied to identify microRNAs (miRNAs) differentially expressed in 47 samples of visceral (VAT) and subcutaneous (SAT) adipose tissues from normal-weight (N), obese (O) and obese after surgery-induced weight loss (PO) individuals. Subsequently miRNA expression was validated by real-time PCR in 197 adipose tissues and bioinformatics analysis performed to identify molecular pathways affected by obesity-related changes in miRNA expression. NGS identified 344 miRNAs expressed in adipose tissues with ≥5 reads per million. Using >2 and <−2 fold change as cut-offs we showed that the expression of 54 miRNAs differed significantly between VAT-O and SAT-O. Equally, between SAT-O and SAT-N, the expression of 20 miRNAs differed significantly, between SAT-PO and SAT-N the expression of 79 miRNAs differed significantly, and between SAT-PO and SAT-O, the expression of 61 miRNAs differed significantly. Ontological analyses disclosed several molecular pathways regulated by these miRNAs in adipose tissue. NGS-based miRNome analysis characterized changes of the miRNA profile of adipose tissue, which are associated with changes of weight possibly responsible for a differential regulation of molecular pathways in adipose tissue when the individual is obese and after the individual has lost weight.

## 1. Introduction

Studies on the pathogenesis of obesity-related complications point to adipose tissue as the main source of mediators involved in pathological changes of the whole-body function. Transcriptome analyses confirmed substantial aberrations in gene expression in the adipose tissues of obese subjects compared to normal-weight individuals [1,2,3,4]; however, molecular mechanisms underlying this phenomenon remain largely unknown.

In recent years there has been great progress in understanding the role of microRNA (miRNA) in the regulation of the expression of various genes, including those related to adipocyte differentiation and function. miRNAs implicated in adipogenesis and adipocyte metabolism were found to be differentially expressed in adipose tissue from obese subjects and from normal-weight controls [1,5,6,7,8] as well as in different adipose tissue depots [9]. However, these studies were performed with microarrays covering only canonical miRNA sequences deposited in databases. Next generation sequencing (NGS) allows for the identification of novel isoforms of miRNA, and has been successfully used for the analysis of miRNomes in other tissues [10,11].

Precise characterization of miRNome may serve as a basis for identification of cellular functions and pathways disturbed in the course of the investigated pathology [12]. Therefore, we have applied NGS for the identification of miRNAs, the expression of which varies between different adipose tissue depots of obese individuals before and after weight loss, as well as between obese and normal-weight individuals. We have also performed bioinformatics analysis in order to identify gene targets and, subsequently, pathways affected by obesity-related changes in miRNA expression in adipose tissue.

## 2. Results

### 2.1. miRNAs Expression in Adipose Tissue

NGS identified 344 mature miRNAs expressed in adipose tissues of obese, normal-weight and obese-after-weight-loss individuals, for which reads per million (RPM) were ≥5 in at least 50% of samples within any of the studied groups. Comparison between the analyzed groups revealed significant differences in expression of several miRNAs. Initial analysis showed that the mean expressions of the investigated genes did not differ in adipose tissues of males and females; therefore, all analyses were performed for both sexes together.

miRNAs are deregulated in adipose tissue of obese individuals. Among these miRNAs, the expression of 20 differed between subcutaneous adipose tissue (SAT) samples from obese (O) and normal-weight (N) individuals at the significance level of False Discovery Rate (FDR) <0.05, including 19 miRNAs up-regulated (O/N fold change > 2, Table 1, columns 2 and 3) and 1 down-regulated in SAT-O vs. SAT-N. The most up-regulated miRNAs were hsa-miR-146b-3p, hsa-miR-146b-5p, hsa-miR-223-3p, hsa-miR-450b-5p and hsa-miR-22-3p, while hsa-miR-205-5p was deeply down-regulated.

Furthermore, the expression of only one miRNA, hsa-miR-424-3p, was significantly up-regulated in visceral adipose tissue of obese subjects (VAT-O) compared to VAT-N (O/N fold change = 2.63 in NGS). None of the miRNAs down-regulated in VAT-O vs. VAT-N passed the cut-off criteria of statistical significance (FDR < 0.05).

Obesity was associated with a significantly higher expression of 47 miRNAs including the most up-regulated hsa-miR-31-5p, hsa-miR-200a-3p, hsa-miR-200b-3p, hsa-miR-204-5p, hsa-miR-429 and hsa-miR-493-3p; it was also associated with a lower expression of seven miRNAs: hsa-miR-23a-5p, hsa-miR-27a-5p, hsa-miR-96-5p, hsa-miR-183-5p, hsa-miR-196a-5p, hsa-miR-486-5p and hsa-miR-615-3p in VAT-O compared to SAT-O (Table 2). In contrast, no significant differences in miRNAs expression between VAT-N and SAT-N were observed in normal-weight individuals.

### 2.2. Weight Loss Is Associated with Change of the SAT miRNA Profile

Next, we analyzed changes in the miRNA profile associated with weight loss. Expression of 61 miRNAs differed significantly between SAT samples obtained from obese subjects before (O) and after weight loss (PO) with a fold change at least >2 or <−2 (Table 1, columns 4 and 5). The expression of three miRNAs: hsa-miR-196a-5p, hsa-miR-483-3p and has-miR-1260b was higher in SAT-PO compared to SAT-O, and the expression of 58 miRNAs, including hsa-miR-96-5p, hsa-miR-144-3p, hsa-miR-182-5p, hsa-miR-183-5p, hsa-miR-223-3p and hsa-miR-486-3p, was lower in SAT-PO than in SAT-O.

A subgroup of miRNAs, namely hsa-miR-146b-3p, hsa-miR-146b-5p, hsa-miR-223-3p, hsa-miR-223-5p and hsa-miR-941, the expression of which was significantly decreased after weight loss, were also identified as up-regulated in SAT-O compared to SAT-N.

### 2.3. miRNA Profile in Adipose Tissue after Weight Loss Differs from That in Adipose Tissue of Normal-Weight Individuals

Finally, we addressed the question as to whether miRNA expression profile differed between SAT samples obtained from the obese subjects after weight loss compared to normal-weight individuals. We found that the level of 79 miRNAs was significantly different, with a fold change at least >2 or <−2 (Table 1, columns 6 and 7). At the top of the list of the 42 miRNAs with lower expression in SAT-PO than in SAT-N were hsa-miR-96-5p, hsa-miR-144-3p, hsa-miR-183-5p, hsa-miR-205-5p, hsa-miR-451a, hsa-miR-486-3p and -5p, while hsa-miR-95, hsa-miR-196a-5p, hsa-miR-615-3p and hsa-miR-1260b headed the list of 37 miRNA with higher expression in SAT-PO than in SAT-N.

Three miRNAs, hsa-miR-27a-5p, hsa-miR-450b-5p and hsa-miR-628-5p, with a higher expression in SAT-O than in SAT-N, remained increased in SAT-PO, while the expression of hsa-miR-205-5p remained reduced. Out of five miRNAs whose expression was significantly increased in SAT-O compared to SAT-N and subsequently decreased after weight loss (hsa-miR-146b-3p, hsa-miR-146b-5p, hsa-miR-223-3p, hsa-miR-223-5p and hsa-miR-941) only hsa-miR-223-3p and -5p had their levels after weight loss significantly lower than in normal-weight subjects.

### 2.4. Real Time PCR Verification of the NGS Results

Real-time PCR (RT-PCR) positively confirmed the obesity-associated expression changes of the selected miRNAs detected by NGS (Table 1 and Table 2). On the other hand, out of three miRNAs with the most stable expression in NGS, only hsa-miR-374a-5p had comparable levels in adipose tissues regardless of weight and weight changes when assessed by real-time PCR, while hsa-miR-214-3p and hsa-miR-342-3p levels were significantly higher in SAT-PO than in SAT-O, indicating that they cannot be used as internal control for miRNA testing in adipose tissue.

### 2.5. Identification of Target Genes Regulated by Differentially Expressed miRNAs in Adipose Tissues

Using the MirWalk and MirTarBase programs, we searched for genes regulated by miRNAs whose expression in adipose tissue was significantly (fold change at least >2 or <−2) affected by obesity and weight loss. We identified 9932 putative gene targets for miRNAs differentially expressed in SAT-O and SAT-N, 16158 targets for miRNAs differentially expressed in SAT-PO and SAT-O, 17186 targets of miRNAs whose levels differed significantly between SAT-PO and SAT-N, and 15565 targets regulated by miRNAs differentially expressed in VAT-O and SAT-O. Analysis of targets was performed separately for each abovementioned pair of tissues. Notably, targets with a known function in obesity (Table 3) were among those with the highest (>10) number of binding sites for miRNAs characterized by differential expression.

Next, we used our estimation method described in the Methods section to evaluate the direction of the expression changes of 10 putative targets that might have been affected by the obesity-related changes in miRNA levels. For this analysis we selected *BMPR2* (encoding bone morphogenic protein receptor 2), *CABP4* (encoding calcium binding protein 4), *CFL2* (encoding cofilin 2), *DBT* (encoding lipoamide acyltransferase component of branched-chain alpha-keto acid dehydrogenase complex), *DISC1* (encoding disrupted in schizophrenia 1), *DOK1* (encoding docking protein 1), *FOXP1* (encoding forkhead box protein P1), *IGF1R* (encoding insulin-like growth factor receptor 1), *MTMR12* (encoding myotubularin related protein 12) and *TRIM14* (encoding tripartite motif-containing protein 14) whose expression, based on the miRNA analysis, should be affected in all analyzed pairs of tissues. In order to establish the relevance of the above analysis, we measured the expression of these targets by real-time PCR. We found that in each case the direction of expression changes predicted by our estimation method agreed with the experimental data (Table S1).

**Figure 1:**
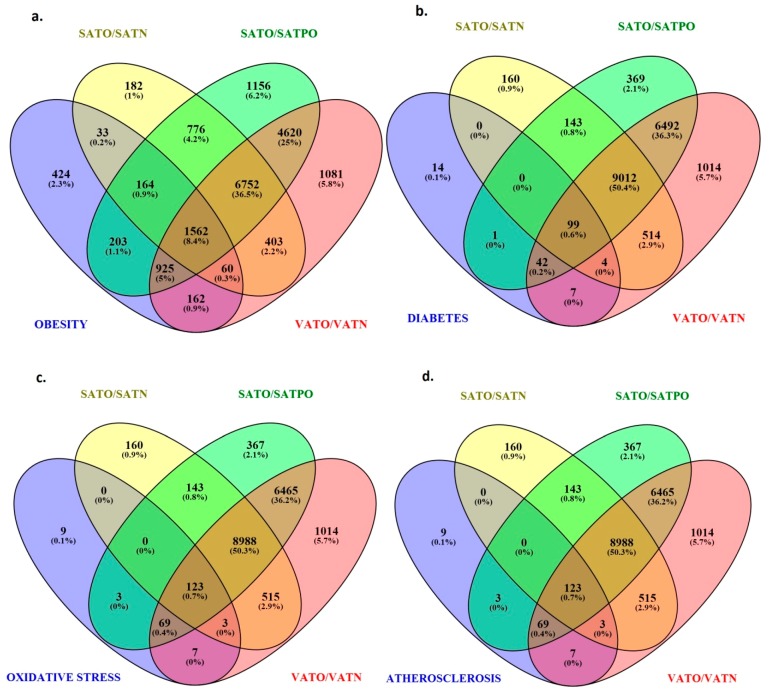
Venn’s diagrams showing involvement of genes regulated by miRNAs differentially expressed in subcutaneous (SAT) and visceral (VAT) adipose tissues from obese (O), normal weight (N), obese after weight-loss (PO) individuals in pathogenesis of obesity (**a**), diabetes (**b**), oxidative stress (**c**) and atherosclerosis (**d**).

Apart from obesity-related genes (Figure 1a), among potential targets for miRNAs differentially expressed in the investigated tissues were also those associated with obesity-related complications including diabetes (Figure 1b), oxidative stress (Figure 1c) and atherosclerosis (Figure 1d).

### 2.6. Molecular Pathways Regulated by Differentially Expressed miRNAs

To identify the molecular pathways associated with a change in the obesity-related miRNome, we employed the PANTHER Classification System. When potentially affected targets were taken into account, 49 pathways were indicated (a summary of the most relevant results is presented in Table 4), some of them overrepresented, some underrepresented, suggesting that obesity is associated with either greater or lower than expected, respectively, regulation of a given pathway by these miRNAs. Among the pathways, only three passed the Bonferroni correction (Table 4).

## 3. Discussion

In this work, we present the results of a comprehensive analysis of adipose tissue miRNome changes associated with excess adiposity. We showed that some obesity-related changes in adipose tissue miRNome do not disappear after weight loss. Consequently, based on in silico analysis, we identified molecular pathways differentially regulated in adipose tissue from normal-weight and obese subjects before and after weight loss.

Among the miRNAs up-regulated in the SAT of obese subjects compared to the SAT of normal-weight controls were those with proadipogenic (e.g., hsa-miR-21-5p, hsa-miR-146b-3p and hsa-miR-450a-5p) as well as those with antiadipogenic properties (hsa-miR-23a-5p and hsa-miR-27a-5p), supporting the notion that obesity is associated with dysregulation of adipogenesis [13]. Apart from hsa-miR-21 and hsa-miR-146b, none of these miRNAs has been previously reported to be up-regulated in SAT-O [12,14,15]. Notably, hsa-miR-450 was found to be down-regulated in adipocyte cell lines isolated from obese patients [7]. This discrepancy could have resulted from the fact that the microarray technique used by Ortega et al. did not distinguish hsa-miR-450 isoforms. Other miRNAs up-regulated in SAT-O were those involved in the regulation of the immune response, acting as suppressors (e.g., hsa-miR-223-3p) or activators (e.g., hsa-miR-146b-5p) of inflammation [12,16,17]. Ontological analysis pointed to an overrepresentation of inflammatory pathways mediated by chemokine and cytokine signaling in SAT-O as compared to SAT-N (Table 4). “Overrepresentation” refers to the increased regulation of a given pathway, but does not firmly indicate whether it is up- or down-regulated; this might only be estimated from the level of expression of miRNAs regulating a given pathway. Nevertheless, literature data point to an increased proinflammatory activity of adipose tissue in obese individuals [18,19]. Other molecular pathways overrepresented in adipose tissue in obesity were those associated with endothelin signaling, apoptosis and p53 action. Experimental data suggest that obesity is associated with an increased endothelin concentration and the pro-apoptotic phenotype of adipose tissue [20,21,22]. In obesity, the beta-2 adrenergic receptor signaling pathway was found to be underrepresented in SAT, and our previous findings point to the lower expression of genes encoding adrenergic beta receptors in adipose tissues of obese individuals compared to tissues obtained from normal-weight subjects [23]. We found hsa-miR-205-5p to be the only gene deeply down-regulated in SAT-O compared to SAT-N. This miRNA has not been previously reported as associated with obesity, however among its targets are those involved in the pathogenesis of obesity-related complications, e.g., genes encoding interleukins (*IL-6*, *IL-17B* and *IL-22*), apolipoproteins (*APOA5*) and their receptors (*APOBR*). Down-regulation of this miRNA in the adipose tissue of obese individuals clarifies one of the mechanisms of obesity-related low-grade inflammation and lipid disturbances [24].

Using the microarray technique, Capobianco et al. identified several miRNAs differentially expressed in VAT of obese and normal-weight individuals [25]. However in the NGS analysis, the only miRNA significantly up-regulated in VAT-O compared to VAT-N was hsa-miR-424-3p that acts as a regulator of the nuclear factor IA (NFIA), a transcription factor required for proper adipocyte differentiation and lipid droplet formation. Therefore, its down-regulation may contribute to adipose tissue dysfunction [26].

Loss of weight was associated with significant changes in the adipose tissue miRNA profile. These changes concerned, among others, the balance between miRNAs involved in the regulation of adipogenesis. Notably, the expression of only one adipogenesis-related miRNA, hsa-miR-146b-3p, that was higher in SAT-O compared to SAT-N, decreased in SAT-PO to the level observed in SAT-N. In addition, we observed a decreased expression of several proadipogenic miRNAs, namely hsa-miR-15a-5p, hsa-miR-107, hsa-miR-143-5p, hsa-miR-194-5p and hsa-miR-210, and an increased expression of the antiadipogenic hsa-miR-196a-5p [13] in SAT-PO vs. SAT-O, but their expression did not return to the levels observed in SAT-N, suggesting that they may represent permanent miRNome changes induced by obesity. Surprisingly, the levels of hsa-miR-18a-3p and hsa-miR-130b-3p, described previously as antiadipogenic, were decreased in SAT-PO [13,27]. In the study by Ortega et al. the expression of hsa-miR-130b was also decreased in adipocytes isolated from the SAT of obese subjects after weight loss; however, as mentioned above, the assay applied by this author did not distinguish between the two hsa-miR-130b isoforms [8]. Compared to SAT-O, SAT-PO was also characterized by a different expression of several miRNAs involved in the regulation of the immune response and ontological analyses pointed to an underrepresentation of inflammatory pathways in post-bariatric SAT. Among other miRNAs with a lower expression in SAT-PO were those crucial for lipid metabolism, such as hsa-miR-144-3p involved in the decrease of HDL formation [28], for insulin signaling such as hsa-miR-96-5p that represses expression of the gene encoding insulin receptor *INSR* [29], for ageing such as hsa-miR-141-3p that accelerates the ageing of human mesenchymal cells [30], and for those promoting the development of cardiovascular and neurodegenerative diseases, such as hsa-miR-3615 and hsa-miR-15b-5p [31,32]. Therefore, the decreased expression of these genes indicates one of the mechanisms by which loss of weight lowers the risk of obesity-associated complications. Loss of weight was also associated with a lower expression of several miRNAs involved in the regulation of oncogenesis, while ontological analyses suggested overrepresentation of pathways involved in apoptosis signaling and p53-action in SAT-PO. Notably, what also distinguished SAT-PO from SAT-N, was the overrepresentation of signaling transmitted *via* cholecystokinine receptors (CCKR) which increase sympathetic nerve activity in brown adipose tissue, enhancing its termogenic activity [33]. To sum up, the miRNome of SAT after loss of weight, despite positive changes compared to the SAT of the obese, still differed from that of normal-weight subjects, suggesting that even after the normalization of body mass index (BMI), a period of obesity leaves a long-lasting imprint on the metabolism of adipose tissue regarding control of functions such as adipogenesis, lipid metabolism, the immune response, insulin signaling, atherosclerosis, ageing, cardiovascular diseases, neurodegenerative disorders and oncogenesis [13,28,29,30,31,32].

Finally, we found that while in normal-weight individuals there was no significant difference in miRNA levels between visceral and subcutaneous adipose tissue, obesity was associated with pronounced differences in the miRNomes of these two depots (Table 2). Consequently, ontological analyses identified several molecular pathways differentially regulated in the VAT and SAT of obese subjects. VAT was characterized by the overrepresentation of pathways associated with inflammation and interleukin signaling. Interpretation of this finding is somewhat difficult since some gene expression analyses pointed to increased pro-inflammatory activity of VAT [1], while other authors obtained the opposite results [19,34]. One of the pathways underrepresented in VAT was that related to presenilin action. Activation of this pathway was detected in transgenic mice genetically predisposed to the development of dementia, in which bodyweight gain and hyperglycemia resulted in increased serum β-amyloid levels [35] In turn, a distinct regulation of pathways associated with glycolysis and WNT (wingless-type MMTV integration site family members) signaling in VAT-O vs. SAT-O, may reflect different potentials of these depots to accumulate excess fat in obese subjects [36]. WNTs are glycoproteins involved in control of cell proliferation and survival and in the context of adipose tissue, WNT canonical signaling pathways were found to restrain differentiation of mesenchymal stem cells towards adipocytes [37].

In summary, a comprehensive, NGS-based miRNome analysis revealed significant differences in miRNA levels between different adipose tissue depots originating from obese and normal-weight individuals, and characterized changes in miRNA profile resulting from weight loss. Bioinformatics tools applied to detect genes and pathways affected by changes in miRNA levels identified pathways involved in the regulation of inflammation, cytokine signaling, adrenergic receptors action and apoptosis. These findings constitute the basis for subsequent functional studies on the pathogenesis of obesity and related complications.

## 4. Materials and Methods

### 4.1. Study Groups

Pairs of visceral (VAT) and subcutaneous (SAT) adipose tissues were obtained from 58 obese patients (O, body mass index (BMI) > 40 kg/m^2^) during bariatric surgery, as described previously [38]. Fifty-five control tissues were collected from normal-weight individuals (N, BMI 20–24.9 kg/m^2^) undergoing elective cholecystectomy (24 samples of VAT and 24 samples of SAT) or operated for inguinal hernia (seven samples of SAT). Nineteen additional samples of SAT were collected from formerly obese subjects about two years after surgery-induced weight loss (PO, BMI 24.3–29.5 kg/m^2^). VAT samples from the PO subjects were unavailable since post-bariatric surgery regards only skin fold removal and is not associated with abdominal cavity opening. Basic clinical characteristics of study participants are summarized in Table S2. The project was approved by the Bioethics Committee of the Medical University of Warsaw, and written informed consent for participation in this study was obtained from all participants.

### 4.2. miRNA Expression Analysis by Next Generation Sequencing

After collection, all adipose tissue samples were immediately frozen at −80 °C, homogenized in liquid nitrogen and stored again at −80 °C. The previously described methods [20] were used for total RNA isolation. RNA integrity was assessed using an Agilent 2100 Bioanalyzer (Agilent Technology, Santa Clara, CA, USA). For NGS, only samples with the highest quality and integrity (RNA integrity number (RIN) > 8) were selected, so this analysis was performed on 44 tissues: 10 pairs of VAT and SAT from the obese study participants (O, eight women, two men), seven pairs of VAT and SAT from normal-weight subjects (N, six women, one man) and 10 SAT samples from individuals after surgery-induced weight loss (PO, eight women, two men).

A small fraction of RNA was isolated, sequenced and analyzed as described previously [10]. Data obtained for each sample were normalized using the RPM normalization according to the formula: RPM = (N_ref_/N_all_) × 10^6^, where N_ref_ is the number of reads mapped to the miRNA reference and N_all_ is the total number of reads mapped to the sample.

### 4.3. Validation of miRNA Expression by Real-Time PCR

The direction of the NGS-detected obesity-associated expression changes was established for 19 miRNAs. For this analysis, we selected 16 miRNAs for which NGS revealed the most significant differences in their expression between the investigated tissues (hsa-miR-22-3p, hsa-miR-96-5p, hsa-miR-125a-5p, hsa-miR-125b-5p, hsa-miR-146b-3p, hsa-miR-183-5p, hsa-miR-193a-3p, hsa-miR-193b-5p, hsa-miR-194-5p, hsa-miR-196a-5p, hsa-miR-205-5p, hsa-miR-223-3p, hsa-miR-424-3p, hsa-miR-450b-5p, hsa-miR-486-5p and hsa-miR-1260b). We also assessed levels of three miRNAs with the most stable expression in NGS (hsa-miR-214-3p, hsa-miR-342-3p and hsa-miR-374a-5p). The analysis of miRNAs expression was performed with the miRCURY LNA™ Universal RT microRNA PCR system (Exiqon, Vedbaek, Denmark) according to the manufacturer’s protocol, as described previously [38]. Reactions were performed in triplicate using RNA from 197 adipose tissues. The results were normalized against the miR-103a-3p expression, the recommended control miRNA for the adipose tissue [2]. The RNA spike-ins provided by the manufacturer, were used to control the quality of RNA isolation and of cDNA synthesis, and as an inter-plate calibrator.

### 4.4. Identification of miRNA Target Genes and Their Molecular Pathways

The MirWalk (http://zmf.umm.uni-heidelberg.de/apps/zmf/mirwalk2) and MirTarBase (http://mirtarbase.mbc.nctu.edu.tw) programs were used to identify target genes regulated by miRNAs with differential expression [39,40,41,42].

Putative target genes were first identified by miRWalk which utilizes a restrictive algorithm to predict mRNA-miRNA interaction sites by comparing the complete nuclear genes and mitochondrial DNA sequences with miRNA 7-nucleotide seed region and by calculating the probability distribution of random matches of a subsequence from the 5′ end of miRNA sequence, using the Poisson distribution. Next, the program compared the obtained results with results from other popular prediction programs: DIANA-microT, miRanda, miRDB, PicTar, Probability of Interaction by Target Accessibility (PITA), RNA22, RNAhybrid and TargetScan/TargetScanS. This analysis was complemented by data from the Advanced Search tool from MirTarBase containing interaction sites that had been verified by functional tests (reporter assay, immunoblot, qPCR, microarray, protein stable isotope labeling with amino acids in cell culture (pSILAC), cross-linking immunoprecipitation followed by sequencing (CLIP-seq)).

A rough estimation of the direction of target gene expression changes was obtained as follows: when miRNA had a lower expression in a given tissue than in another tissue with which it was compared, we assigned the value of +1 to the expression of its target gene and opposite, when miRNA expression was higher than in the other tissue, we assigned the value of −1. For each gene, the respective values were summarized. When the sum had a positive value, we considered the expression of the gene to be increased, while a negative value indicated gene suppression. Results of this estimation were verified for 10 genes (enlisted in the Results section) by real-time PCR as described previously, with specific primers enlisted in Table S3 [38].

Subsequently, target genes were analyzed with the PANTHER (Protein ANalysis THrough Evolutionary Relationships) Classification System v. 11.0 (Paul Thomas Lab, Los Angeles, CA, USA) combining gene functions, ontology and pathways [43]. Based on this analysis, molecular pathways that were regulated by miRNAs with differential expression were identified. Differences in the regulation of pathways were presented as fold enrichment. Fold enrichment values >1 corresponded to the overrepresentation (increased regulation) of the particular pathway, while values <1 to its underrepresentation (decreased regulation) as compared to control tissue from a given pair of tissues.

### 4.5. Statistical Analysis

NGS analysis and selection of differentially expressed miRNAs were performed using the R/Bioconductor (Roswell Park Cancer Institute, Buffalo, NY, USA) software with paired Welch *t*-test. False discovery rate (FDR) was used to assess the multiple testing errors.

Analysis regarding RT-PCR data on miRNA and mRNA expression was performed with the Statistica Version 7.0 software package (StatSoft, Tulsa, OK, USA) using Student’s t or Mann-Whitney’s *U* test when appropriate. Assessment of normality of the distribution was performed with the Shapiro-Wilk test.

To identify molecular pathways, the sets of genes were analyzed using the binomial overrepresentation test with Bonferroni correction from the Statistical Enrichment Test tool included in the PANTHER Classification System program v. 11.0 (Paul Thomas Lab, Los Angeles, CA, USA).

## Figures and Tables

**Table 1:** Fold changes of microRNAs (miRNAs) differentially expressed in subcutaneous adipose tissue (SAT) of obese individuals before (O) and after weight loss (PO), as well as of normal-weight subjects (N) assessed by the next generation sequencing (NGS) and real-time PCR methods.

	SAT-O vs. SAT-N		SAT-PO vs. SAT-O		SAT-PO vs. SAT-N	
	NGS	Real-Time PCR	NGS	Real-Time PCR	NGS	Real-Time PCR
1	2	3	4	5	6	7
**Proadipogenic**						
hsa-miR-15a-5p *			−2.992		−2.286	
hsa-miR-21-5p *	2.728					
hsa-miR-107			−3.057		−3.734	
hsa-miR-143-5p *			−2.126			
hsa-miR-146b-3p	7.253	2.710	−4.722	−2.972		
hsa-miR-150-5p *					2.128	
hsa-miR-194-5p			−3.029	−3.645	−2.109	−7.00
hsa-miR-199b-5p					2.803	
hsa-miR-210			−2.130		−2.617	
hsa-miR-214-5p *					2.266	
hsa-miR-335-3p					2.574	
hsa-miR-450a-5p	2.282				2.100	
**Antiadipogenic**						
hsa-miR-18a-3p			−3.703		−3.699	
hsa-miR-23a-5p	2.093					
hsa-miR-27a-5p *	2.363				2.512	
hsa-miR-29a-3p *					2.010	
hsa-miR-130b-3p			−4.053		−2.787	
hsa-miR-196a-5p			4.031	3.162	4.047	2.97
**Involved in adipocyte browning**						
hsa-miR-125b-5p					2.247	2.01
**Involved in inflammation and immune response**						
hsa-miR-15b-3p *			−5.090		−6.432	
hsa-miR-16-2-3p *			−7.972		−6.263	
hsa-miR-20b-5p *			−2.936		−3.518	
hsa-miR-22-3p *	3.001	2.011				
hsa-miR-32-5p *			−2.452			
hsa-miR-92b-3p *					2.228	
hsa-miR-93-5p			−3.602		−3.358	
hsa-miR-106a-5p *			−3.470		−4.302	
hsa-miR-122-5p *			−5.792			
hsa-miR-125a-5p *					2.150	1.96
hsa-miR-125b-1-3p *	2.063					
hsa-miR-142-3p *			−5.215			
hsa-miR-142-5p *			−6.087		−5.233	
hsa-miR-146b-5p *	5.077		−3.986			
hsa-miR-182-5p *			−15.039			
hsa-miR-223-3p *	5.804	3.041	−27.825	−8.036	−4.794	−2.72
hsa-miR-3200-3p			−3.103		−3.802	
**Involved in lipid metabolism**						
hsa-miR-106b-5p *			−3.778		−3.984	
hsa-miR-144-3p *			−11.390		−14.187	
hsa-miR-185-5p			−2.836		−3.303	
**Involved in atherosclerosis**						
hsa-miR-365a-3p					2.559	
hsa-miR-365b-3p					2.559	
**Involved in insulin signaling**						
hsa-miR-96-5p			−22.917	−9.251	−13.913	−15.01
hsa-miR-128			−2.418			
**Involved in oncogenesis**						
hsa-let-7e-3p					2.100	
hsa-miR-16-5p			−2.755			
hsa-miR-17-3p			−2.066			
hsa-miR-18a-5p			−2.410		−4.324	
hsa-miR-18b-5p			−4.240		−6.464	
hsa-miR-25-3p			−2.289		−2.448	
hsa-miR-28-5p					2.086	
hsa-miR-95					3.118	
hsa-miR-106b-3p			−3.636			
hsa-miR-144-5p			−8.083		−8.337	
hsa-miR-183-5p			−14.823	−3.723	−20.421	−7.62
hsa-miR-192-5p			−3.234		−3.408	
hsa-miR-193b-5p					2.500	1.97
hsa-miR-205-5p	−10.897	−5.541			−10.030	−10.73
hsa-miR-215	2.015					
hsa-miR-223-5p	2.894		−5.798		−2.003	
hsa-miR-301b	2.972					
hsa-miR-330-3p					2.452	
hsa-miR-340-3p					2.073	
hsa-miR-361-3p					2.504	
hsa-miR-363-3p			−5.212		−4.293	
hsa-miR-423-5p			−2.430			
hsa-miR-424-3p			−2.020			
hsa-miR-425-5p			−3.062		−2.103	
hsa-miR-451a			−9.299		−11.488	
hsa-miR-483-3p			2.681		2.860	
hsa-miR-489					2.440	
hsa-miR-501-3p			−3.074			
hsa-miR-518b	2.705					
hsa-miR-548d-5p			−3.669		−3.766	
hsa-miR-550a-3p			−3.100			
hsa-miR-576-5p			−3.083		−3.587	
hsa-miR-584-5p			−2.741		−2.377	
hsa-miR-615-3p					3.163	
hsa-miR-628-5p	2.569				2.504	
hsa-miR-629-5p			−2.241		−2.261	
hsa-miR-652-3p			−2.807		−2.653	
hsa-miR-671-3p					2.878	
hsa-miR-874					2.070	
hsa-miR-891a	2.984					
hsa-miR-941	2.404		−2.239			
hsa-miR-1260b			2.460	1.524	4.854	2.30
hsa-miR-1271-5p	2.540					
hsa-miR-1285-3p			−2.396			
hsa-miR-3688-3p			−5.070		−5.732	
hsa-miR-4662a-5p					3.410	
hsa-miR-4732-3p			−3.347		−3.124	
hsa-miR-5683					5.557	
hsa-miR-6716-3p					2.481	
**Involved in ageing**						
hsa-miR-141-3p			−5.518		−8.255	
hsa-miR-369-5p					2.554	
**Involved in cardiovascular diseases**						
hsa-miR-589-5p					2.193	
hsa-miR-3615			−5.278		−4.355	
**Involved in neurodegenerative disorders**						
hsa-miR-15b-5p			−2.811		−2.753	
hsa-miR-132-5p	2.921					
hsa-miR-151a-5p					2.228	
hsa-miR-548ay-5p			−3.505		−3.731	
hsa-miR-664a-3p					2.102	
hsa-miR-3607-3p					2.706	
hsa-miR-3909					2.865	
**Unknown function**						
hsa-miR-450b-5p	3.284	3.723			2.605	7.13
hsa-miR-486-3p			−12.206		−11.022	
hsa-miR-486-5p			−9.535	−4.464	−9.118	−8.83

* miRNA also associated with other obesity-related pathologies.

**Table 2:** Fold changes of miRNA differentially expressed in visceral (VAT) and subcutaneous adipose tissue (SAT) of obese (O) individuals assessed by the next generation sequencing (NGS) and the real-time PCR methods.

	VAT-O vs. SAT-O	
	NGS	Real-Time PCR
**Proadipogenic**		
hsa-miR-450a-5p	2.023	
**Antiadipogenic**		
hsa-miR-23a-5p	−3.371	
hsa-miR-27a-5p	−2.512	
hsa-miR-29b-3p	2.471	
hsa-miR-29c-3p	2.084	
hsa-miR-196a-5p	−14.684	−17.21
**Involved in adipogenesis**		
hsa-miR-125b-5p	2.084	1.89
**Involved in adipocytes browning**		
hsa-miR-539-3p	2.127	
**Involved in inflammation and immune response**		
hsa-miR-125a-5p	2.615	1.98
**Insulin signaling**		
hsa-miR-33a-3p	2.283	
hsa-miR-33b-5p	3.170	
hsa-miR-96-5p	−2.887	−2.23
hsa-miR-133a	2.720	
hsa-miR-133b	2.665	
**Involved in oncogenesis**		
hsa-let-7e-3p	2.145	
hsa-miR-31-5p	10.078	
hsa-miR-100-3p	2.979	
hsa-miR-100-5p	2.647	
hsa-miR-101-5p	2.647	
hsa-miR-136-5p	2.562	
hsa-miR-183-5p	−2.780	−1.98
hsa-miR-190a	2.017	
hsa-miR-193a-3p	3.319	2.03
hsa-miR-200a-3p	5.333	
hsa-miR-200b-3p	5.784	
hsa-miR-203a	4.713	
hsa-miR-204-5p	6.024	
hsa-miR-218-5p	2.537	
hsa-miR-337-3p	2.051	
hsa-miR-376c-3p	2.908	
hsa-miR-381-3p	2.405	
hsa-miR-424-5p	2.573	
hsa-miR-429	5.626	
hsa-miR-486-5p	−2.872	−2.67
hsa-miR-487b	2.002	
hsa-miR-493-3p	5.581	
hsa-miR-493-5p	5.259	
hsa-miR-497-5p	2.083	
hsa-miR-532-3p	2.006	
hsa-miR-551b-3p	4.019	
hsa-miR-561-5p	2.275	
hsa-miR-615-3p	−3.246	
hsa-miR-887	2.400	
hsa-miR-1307-5p	2.601	
**Involved in ageing**		
hsa-miR-299-3p	3.240	1.98
**Involved in cardiovascular disorders**		
hsa-miR-423-3p	2.013	
hsa-miR-495-3p	2.702	
**Involved in neurodegenerative disorders**		
hsa-miR-9-5p	2.536	
hsa-miR-101-3p	2.979	
hsa-miR-136-3p	2.436	
hsa-miR-376a-5p	2.099	
hsa-miR-382-3p	2.184	
hsa-miR-485-3p	2.122	
hsa-miR-3607-3p	3.016	

**Table 3:** Common obesity-related targets with the highest (>10) number of binding sites for miRNAs differentially expressed in subcutaneous (SAT) and visceral (VAT) adipose tissues of normal weight (N) and obese before (O) and after surgery (PO) individuals.

SAT-O vs. SAT-N	SAT-O vs. SAT-PO	SAT-N vs. SAT-PO	VAT-O vs. SAT-O
*PPARA*	*PRKAR1A*	*VEGFA*	*ADRB2*
*CASP9*	*CCND1*	*CREB1*	*VEGFA*
*PPARGC1B*	*PTEN*	*BCL2*	*BCL2*
*IGF1R*	*CDKN1A*	*FLT1*	*SCARB1*
*FMR1*	*HMGA2*	*MDM2*	*CDC42*
*SLC26A2*	*HMGA1*	*CCR2*	*PAX8*
*RAD51B*	*XIAP*	*PCDH15*	*SP1*
*MBD4*	*CCND2*	*EXOC7*	*SLC16A1*
*FOXP1*	*SMAD4*	*ARL17A*	*BICD1*
*RNASEL*	*CADM1*	*PARD3*	*CSNK1D*
*CAMTA1*	*SPRED1*	*BICD1*	*MCL1*
*BMPR2*	*MBD1*	*ADRA1A*	*IL1RAP*
*VIPR2*	*HSPA1B*	*TMPO*	*CLTC*
*DBT*	*NOTCH2*	*QKI*	*CELF2*
*CDS1*	*BRWD1*	*DNAJC10*	*ENAH*
*WHSC1L1*	*E2F3*	*ZNF496*	*HSPA13*
*CELF1*	*MAPK9*		*PCDH7*
*DAPK2*	*NRG1*		*HDGF*
*PHF20*	*SLC2A3*		*DYNC1I1*
*CRYL1*	*KIF6*		*PPP1R12B*
*CCDC13*	*HMBOX1*		
	*TPGS2*		

**Table 4:** Selected molecular pathways significantly regulated by differentially expressed miRNAs in subcutaneous (SAT) and visceral (VAT) adipose tissues of normal-weight (N), obese (O) and after weight loss (PO) individuals. Values presented as fold enrichment (values >1 correspond to the overrepresentation of the particular pathway while values <1 to its underrepresentation).

	Fold Enrichment			
	SAT-O vs. SAT-N	SAT-PO vs. SAT-O	SAT-PO vs. SAT-N	VAT-O vs. SAT-O
Pathways corrected *				
Inflammation mediated by chemokine and cytokine signaling pathway (P00031)	1.22	−1.13	−1.14	1.17
Wnt signaling pathway (P00057)				−1.53
CCKR signaling map (P06959)			1.35	
Pathways non corrected **				
Beta2 adrenergic receptor signaling pathway (P04378)	−2.46			
Interleukin signaling pathway (P00036)				1.23
Endothelin signaling pathway (P00019)	1.46			
Apoptosis signaling pathway (P00006)	1.25	1.81	−1.23	−1.47
p53 pathway (P00059)	1.30	1.78	−1.28	−1.68
p53 pathway by glucose deprivation (P04397)	1.63			
Alzheimer disease-presenilin pathway (P00004)	−1.28			−1.47
Glycolysis (P00024)		3.55		−2.64
Cholesterol biosynthesis (P00014)				−2.44

* Pathways that passed the Bonferroni correction; ** Pathways that did not pass the Bonferroni correction.

## Supplementary Materials

Supplementary materials can be found at www.mdpi.com/1422-0067/19/1/66/s1.
