# Supplementary material for: NGS Reveals Molecular Pathways Affected by Obesity and Weight Loss-Related Changes in miRNA Levels in Adipose Tissue
Source: Int J Mol Sci. 2017 Dec 27;19(1):66. doi: 10.3390/ijms19010066 (PMC5796016; doi:10.3390/ijms19010066)
Supplement: Supplementary file 1 [file ijms-19-00066-s001.pdf]

Article

NGS Reveals Molecular Pathways Affected by Obesity and Weight Loss-Related Changes in miRNA Levels in Adipose Tissue

Alina Kuryłowicz [1,*], Zofia Wicik [1], Magdalena Owczarz [1], Marta Izabela Jonas [1], Marta Kotlarek [2], Michał Świerniak [2], Wojciech Lisik [3], Maurycy Jonas [3], Bartłomiej Noszczyk [4] and Monika Puzianowska- Kuźnicka [1,5,*]

Table S1. Direction of expression of 10 selected targets in subcutaneous (SAT) and visceral (VAT) adipose tissue of obese individuals before (O) and after (PO) weight loss as well as of normal-weight subjects (N) assessed predicted by and experimentally measured by the *real-time* PCR method (presented as fold changes).

Gene	SAT-O vs SAT-N		SAT-O vs SAT-PO		SAT-N vs SAT-PO		VAT-O vs SAT-O	
	**predicted**	**measured**	**predicted**	**measured**	**predicted**	**measured**	**predicted**	**measured**
BMPR2	↓	-1.29	↓	-1.00	↓	-1.28	↓	-1.21
CABP4	↓	-1.00	↓	-1.13	↓	-1.62	↓	-1.00
CFL2	↓	-2.92	↓	-1.33	↓	-2.19	↓	-1.55
DBT	↓	-2.27	↓	-1.76	↓	-1.28	↓	-2.41
DISC1	↓	-2.77	↓	-1.21	↓	-2.29	↓	-1.35
DOK1	↓	-1.66	↓	-1.33	↑	1.55	↓	-1.42
FOXP1	↓	-1.5	↓	-1.18	↓	-1.27	↓	-1.18
IGF1R	↓	-1.6	↓	-1.12	↓	-1.41	↓	-2.01
MTMR12	↓	-2.54	↓	-1.4	↓	-1.75	↓	-2.14
TRIM14	↓	-2.03	↓	-4.375	↑	2.15	↓	-2.91

↓: decreased expression; ↑: increased expression

Table S2. Selected clinical and biochemical parameters of study participants.

	**Obese individuals before weight loss**		**Obese individuals after weight loss**		**Normal-weight controls**	
Males/Females	10/48		4/15		9/22	
	Mean±SD	Min-Max	Mean±SD	Min-Max	Mean±SD	Min-Max
Age (years)	41.52 ± 10.31	20-59	41.47 ± 10.27	28-67	43.26 ± 14.93	28-62
Weight (kg)	133.2 ± 17.46	101.7-198	76.11 ± 7.15	68-90	64.62 ± 16.23	56-90
BMI (kg/m^2)	47.37 ± 4.55	40.26-59.26	27.2± 2.35	24.30-29.50	22.95 ± 1.64	20.07-24.96
Weight loss (kg)	-	-	47.8± 10.4	35.20-65.60	-	-
TSH (mIU/l)	1.728 ± 0.326	0.334-3.650	1.583± 0.278	1.12-2.01	1.22 ± 0.18	1.09-1.35
Glucose (mmol/l)	5.56 ± 1.49	3.22-10.16	4.82±0.55	4.12-5.66	4.97 ± 0.53	4.22-5.50
Total cholesterol (mmol/l)	5.20 ± 1.05	3.13-7.87	4.61 ± 0.88	3.52-5.89	4.87 ± 0.36	4.81-5.33
Co-morbidities	N	%	N	%	N	%
Hypertension	35	60.34	6	31.6	none	none
Type 2 diabetes / IGT	26	44.83	3	15.8	none	none
Hyperlipidemia	36	62.09	6	31.6	none	none

BMI: body mass index calculated as weight (kg) divided by height squared (m^2), IGT: impaired glucose tolerance, N: number of patients affected.

Table S3. Primers used for the analysis of expression the selected targets on mRNA level in adipose tissues.

Gene	Gene Bank		Primers	Annealing (°C)
BMPR2	NM_001204	F	5′TTAGTGACTTTGGACTGTCCATGAG3′	60
		R	5′TCTAGCACTTCTGGTGCCATATATCT3′	
CABP4	NM_001300895	F	5′GAACTGGGCCCCGAGGAG3′	60
		R	5′CTCAGCTTTGGGCCTATCAG3′	
CFL2	NM_001243645	F	5′GCTGGAGTGCAGTGTCAAAA3′	60
		R	5′GGAGGTCAGAGGACAGCTTG3′	
DBT	NM_001918	F	5′CTCTCCGTGGACAGGTTGTT3′	60
		R	5′GCTTCCCCACATAGGCAATA3′	
DISC1	NM_001012957	F	5′GCAGCCATGGATAGTTCTGAGA3′	58
		R	5′TGCAGCTGTTGCTACTCAACTCT3′	
DOK1	NM_001197260	F	5′ATGGACGGAGCAGTGATGGA3′	60
		R	5′CCCAGGTCTTCCTCCACCTC3′	
FOXP1	NM_001244808	F	5′CAGATATTGCGCAGAACCAA3′	60
		R	5′GCAAACATTCGTGTGAACCA3′	
IGF1R	NM_000875	F	5′TGCCTTGGTCTCCTTGTC3′	61
		R	5′TTTCCCTGCTTTGATGGTC3′	
MTMR12	NM_001040446	F	5′CAGCCGCTAGTGCTGGTG3′	60
		R	5′GACACGAAGGAGGGCTTG3′	
TRIM14	NM_014788	F	5′GCAGCAGCACATTGACAACA3′	58
		R	5′TCCACGAGGCCCTTAAAGAA3′	

F: forward primer, R: reverse primer.
